# Anterolateral Thigh Flow-Through Flap in Hand Salvage

**Published:** 2013-04-10

**Authors:** Amy F. Kells, Justin M. Broyles, Antonio F. Simoa, Valerae O. Lewis, Justin M. Sacks

**Affiliations:** ^a^Division of Plastic and Reconstructive Surgery, Washington University in St Louis, St Louis, Mo; ^b^Department of Plastic and Reconstructive Surgery, The Johns Hopkins Hospital, Baltimore, Md; ^c^Division of Plastic Surgery, University of Texas Medical Branch, Galveston, Tex; ^d^Department of Orthopaedic Oncology, The University of Texas MD Anderson Cancer Center, Houston, Tex

## Abstract

**Objective:** Hand salvage and reconstruction following trauma and oncologic resection often dictates the use of innovative reconstructive techniques. Preservation of functional anatomy is paramount to success in this clinical setting. Further constraints are placed on the reconstructive surgeon in the setting of the aging US population. We report a case of successful hand salvage in an elderly patient using a free anterolateral thigh flow-through flap. **Methods:** A retrospective chart review was performed on prospectively entered data to examine the case in detail. Indications, radiographs, and follow-up visits were reviewed. A free anterolateral thigh flap was harvested and used to provide soft tissue coverage as well as reconstruction of the palmar arch. **Results:** The free anterolateral thigh flap not only reconstructed the unique soft tissue envelope of the hand but also restored functional vascular anatomy by reconstituting the interrupted superficial palmar arch. The patient had an uneventful hospital course and was discharged without complications. **Conclusions:** The free anterolateral thigh flap is a versatile flap that can be used as an innovative solution for hand salvage where vascular anatomy and soft tissue need to be restored.

Upper extremity reconstruction following surgical extirpation is critical to restoration of both form and function. Hand reconstruction poses unique challenges because of its intricate anatomy and function. Further limitations are placed on the reconstructive options in the elderly patient population. These patients are often reticent to embark on further debilitating procedures. We describe a case, which illustrates a reconstructive option for restoration of both anatomy and function in hand salvage, ideal for the elderly population.

Immediate flap reconstruction following tumor resection is indicated when local tissue is insufficient for direct closure. Multiple flap options exist; however, reconstructive dogma is a tension-free closure and obliteration of dead space with minimal donor site morbidity.^1^ Free tissue transfer can be superior to pedicled flaps following resection of large or recurrent tumors. Free flaps have been proven to reduce infection, induce bony healing, and optimize limb salvage.^1^

The anterolateral thigh (ALT) free flap can provide a large fasciocutaneous skin paddle, ideal for hand and upper extremity reconstruction with minimal donor site morbidity.^2^^,^^3^ Harvesting the flap with 2 or more perforators provides the additional option of dividing the flap into multiple independent skin segments for resurfacing tissues separated by skin.^4^ Furthermore, the ALT flap has vascular anatomy which enables its use as a flow-through flap, giving it the benefit of reconstructing vascular structures while perfusing adjacent soft issue.^3^^,^^5^

In this article, we report a case of a large composite defect of the hand where the free ALT flap was the optimal reconstructive choice for hand salvage. Both soft tissue and vascular anatomy were reconstructed, and the patient maintained a high degree of satisfaction with her repair 6 months postoperatively.

## METHODS

**Figure 1:**
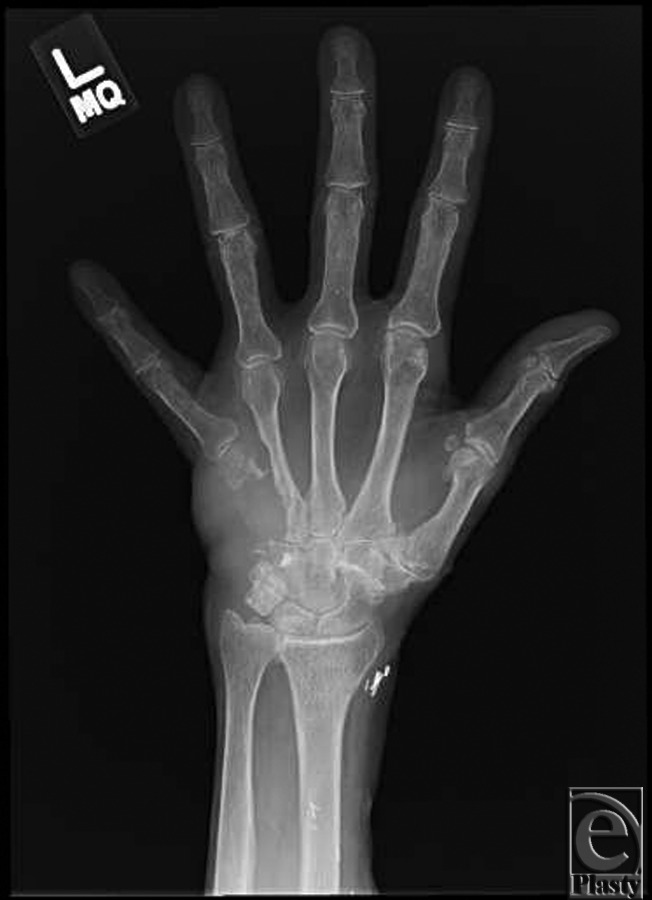
Preoperative radiograph demonstrates a large hypothenar soft tissue mass with osseous destruction of the proximal two thirds of the fifth metacarpal and adjacent cortex of the fourth metacarpal.

A 76-year-old right-hand dominant Caucasian woman with a history of recurrent cutaneous squamous cell carcinoma of the left hand was initially diagnosed in 2002. Prior treatment included multiple Mohs’ procedures of her left hand. The patient did well until she began to experience weakness, recurrent abduction of the fifth finger, and serous drainage from the dorsum of her hand. Radiographs demonstrated osseous involvement, and the patient was referred to the MD Anderson Cancer Center in Houston, Texas, for definitive management (Fig 1).

**Figure 2:**
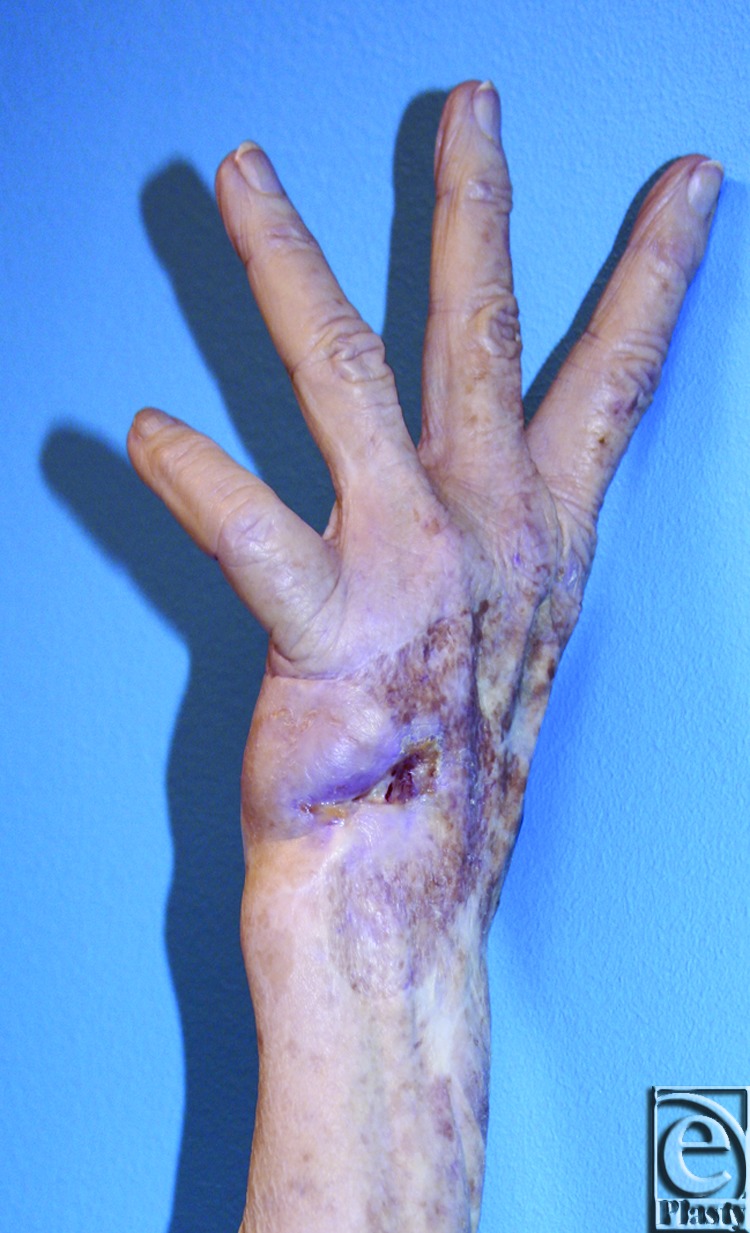
Preoperative imaging demonstrates wound and finger extension.

**Figure 3:**
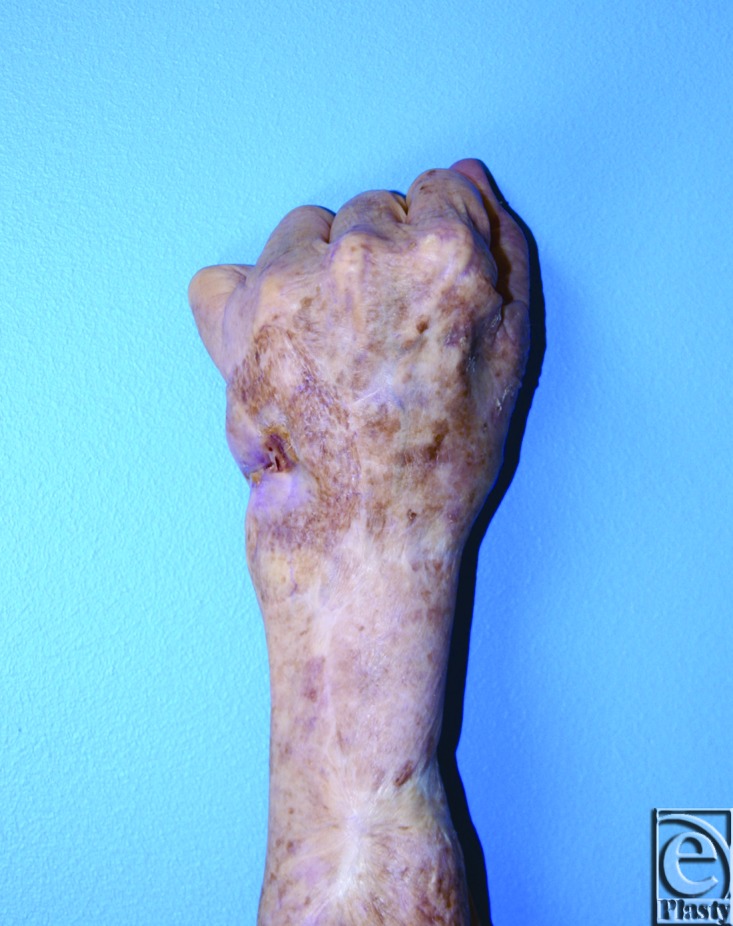
Preoperative imaging demonstrates wound and finger flexion.

Examination of the left hand revealed an open wound, approximately 1 cm in its greatest dimension, on the ulnar aspect of the dorsum of the hand (Figs 2 and 3). The median, ulnar, and radial nerves had preservation of their gross motor function as well as sensibility. Allen test demonstrated an interrupted arch to the left hand with an intact arch on the right. Radiographic examination of the left hand demonstrated a large hypothenar soft tissue mass with osseous destruction of the proximal two thirds of the fifth metacarpal and adjacent ulnar cortex of the fourth metacarpal (Fig 1). Surgical reconstruction was planned in conjunction with excision per the orthopedic oncology service.

**Figure 4:**
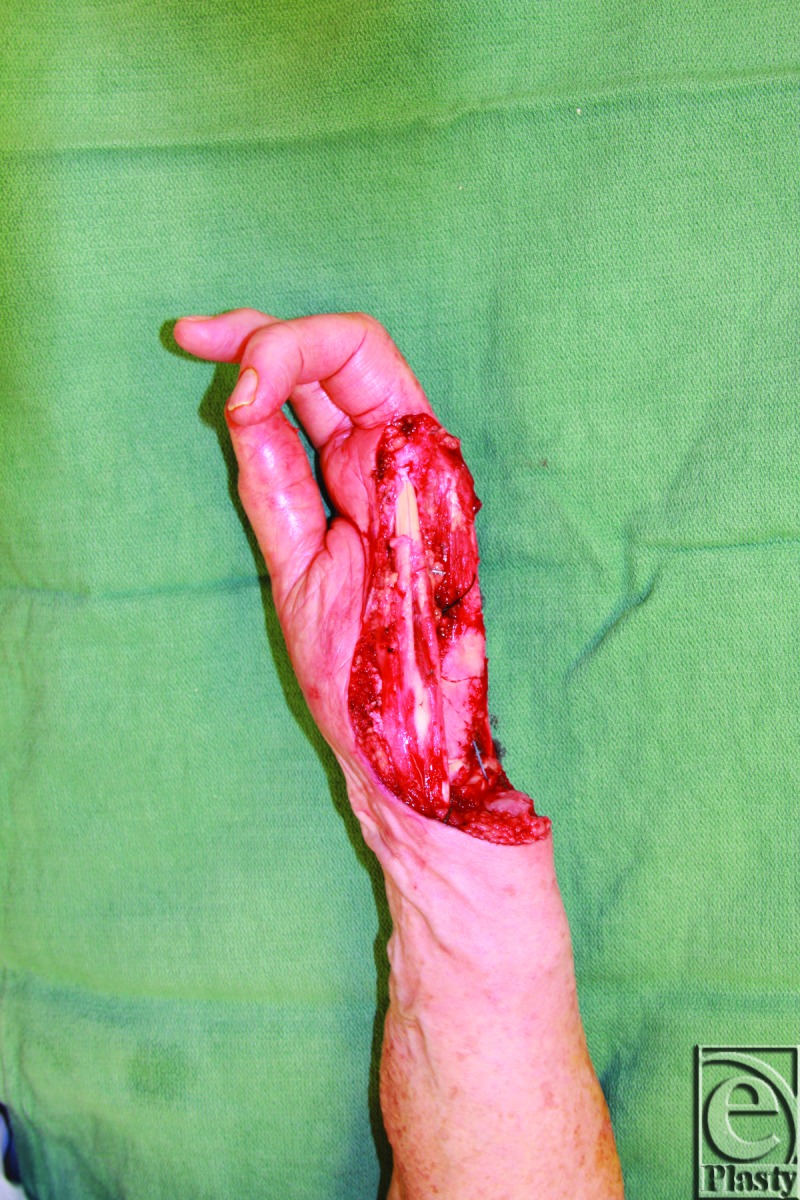
Intraoperative imaging demonstrates extent of soft tissue defect following oncologic resection with amputation of ring and little fingers and segmental resection of the superficial arch of the left hand.

**Figure 5:**
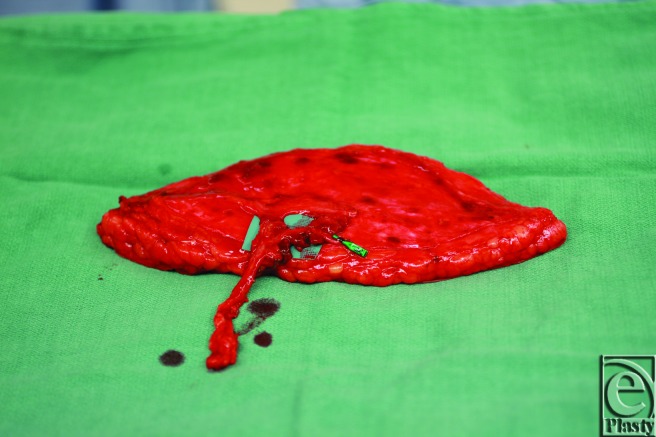
Intraoperative imaging demonstrates the harvested anterolateral thigh flap in situ with 2 perforators from the descending branch of the lateral circumflex femoral artery.

Intraoperatively, following wide local excision, there was a large soft tissue defect of the left hand with small and ring finger amputations (Fig 4). The soft tissue excision left exposure of carpal bones as well as the distal ulna and radius. Margins were negative per pathology. The ulnar artery was included in the resection specimen from just proximal to Guyon's Canal to mid superficial arch. However, the thumb and index and middle fingers maintained good capillary refill and the distal remaining superficial palmar arch had weak retrograde filling. Given the defect size, location, and requirement for vascular reconstruction, the left anterolateral thigh free flap was selected for hand salvage and reconstruction. Dissection and harvest of the left thigh ALT flap was performed as described.^5^ Briefly, 2 perforators were visualized, perforator A and B, at 24 cm and 29 cm, respectively, and dissected to harvest a 2 perforator ALT perforator flap (Fig 5). Following dissection, the flap had good capillary refill and the motor nerves to the thigh muscles were preserved.

Prior to ALT flap harvest, recipient vessels were prepared. The proximal ulnar artery stump was dissected and inflow was assessed as adequate, but the vena comitantes were noted to be insufficient for venous outflow. Thus, a superficial subcutaneous vein was identified and prepared for anastomosis. The ALT flap was harvested and inset in the left hand defect. Flap inflow was established by anastomosis of the ALT pedicle to the ulnar artery in an end-to-end fashion using interrupted 9-0 nylon suture in standard fashion. After documenting good arterial inflow, the flow-through property of the ALT flap was used to reconstruct the superficial palmer arch. The distal end of the lateral circumflex femoral artery was anastomosed to the distal stump of the transected ulnar artery in the palm using interrupted 11-0 nylon sutures. The ALT flap was covered with a sterile bulky dressing and the patient was placed in a volar splint.

## RESULTS

**Figure 6:**
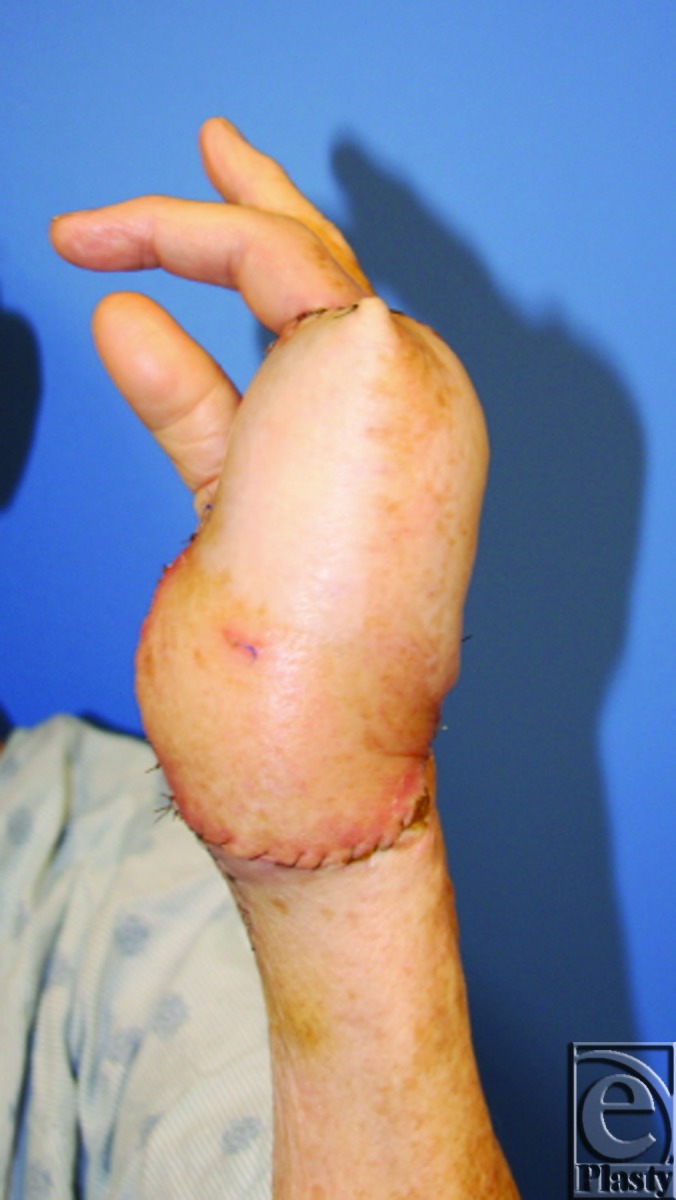
Postoperative imaging at 2 weeks demonstrates a viable anterolateral thigh flap and left hand.

At 2 weeks postoperative follow-up, the patient showed excellent healing of all surgical wounds without complications (Fig 6). The patient was recovering motion of her thumb and index and middle fingers with the assistance of physical and occupational therapy.

**Figure 7:**
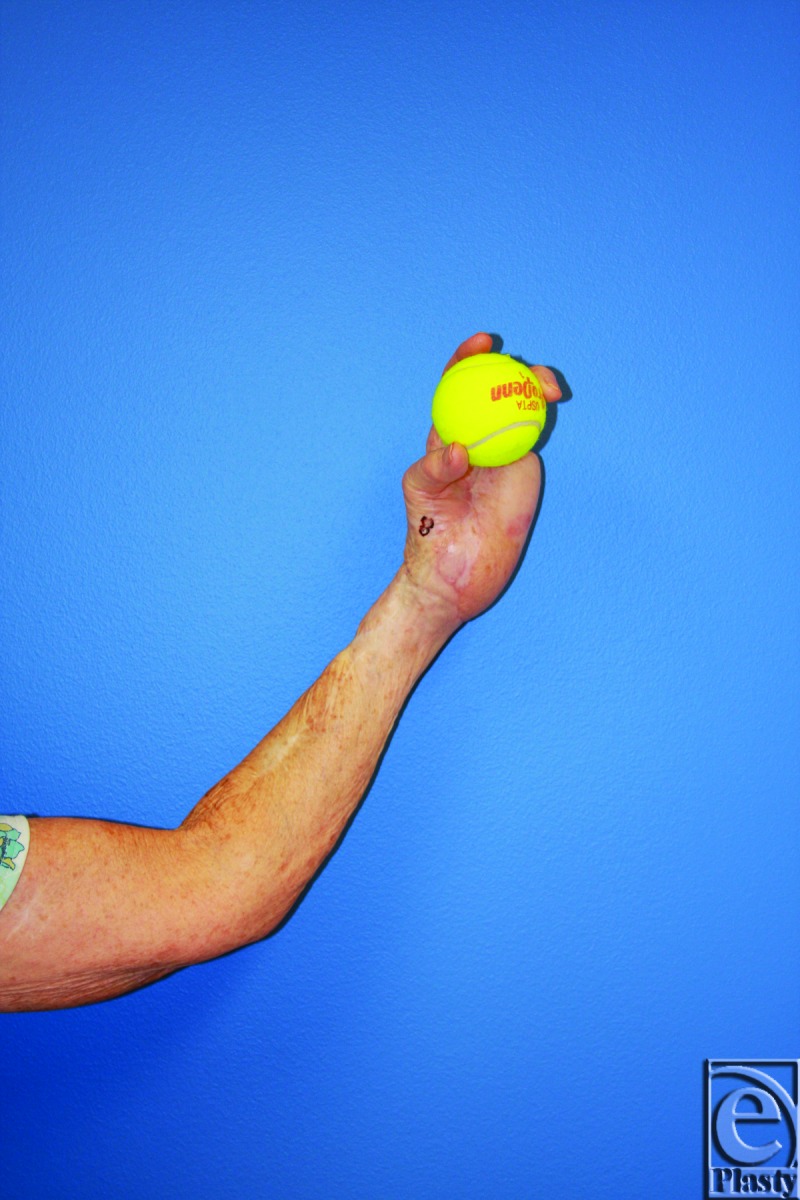
Postoperative imaging at 6 months demonstrates a well-healed anterolateral thigh flap with viable left hand.

**Figure 8:**
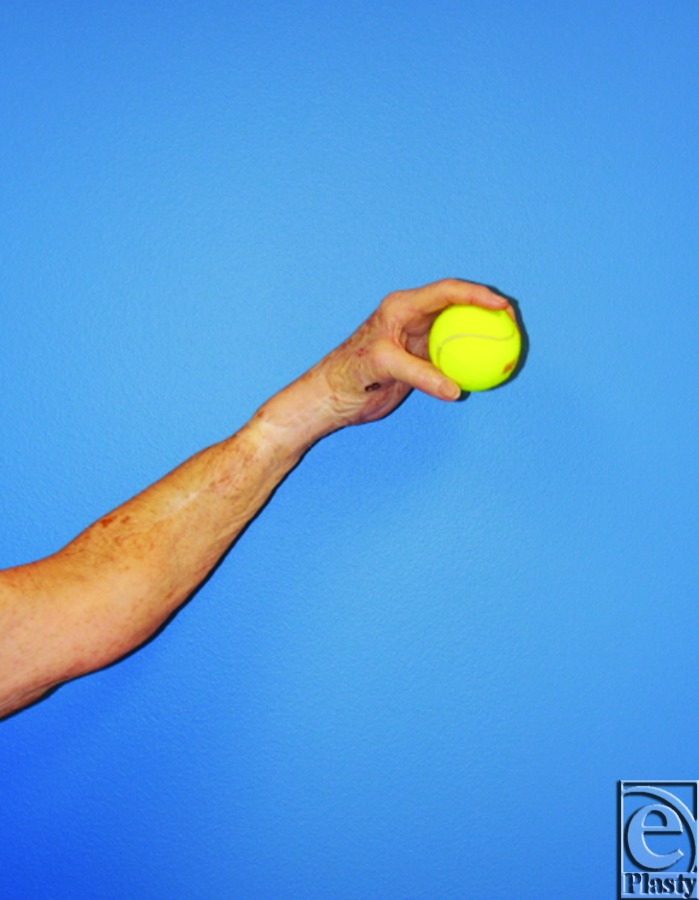
Postoperative imaging at 6 months demonstrates the patient with return to her activities of daily living, which included tennis.

**Figure 9:**
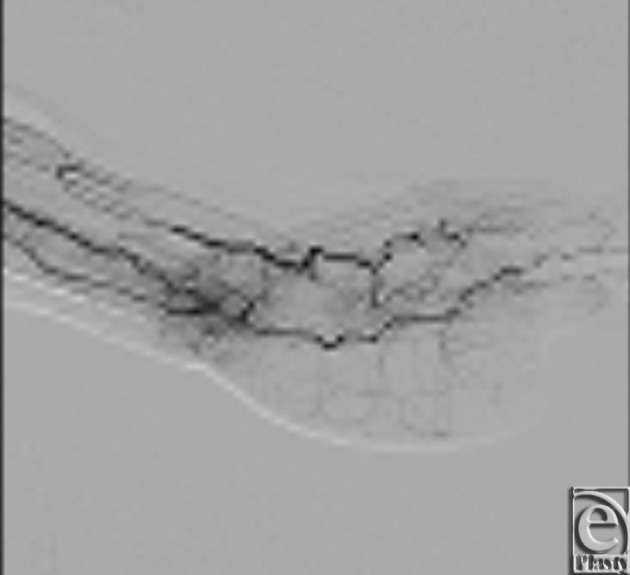
Postoperative angiogram at 6 months, which demonstrates a patent reconstructed superficial palmer arch of the left hand with patency of the flow-through anterolateral thigh flap vessel.

At 6 months follow-up, the patient had recovered increased range of motion of the remaining fingers of her left hand (Figs 7 and 8). The patient reported she had returned to all her previous activities including the ability to play tennis. Angiographic evaluation demonstrated a patent-reconstructed superficial palmer arch of the left hand, with patency of the flow-through ALT flap vessel (Fig 9).

## DISCUSSION

Resection of hand tumors frequently necessitates excision of significant soft-tissue and/or bone. Composite tissue loss necessitates microsurgical techniques, such as nerve or vessel repair as well as a familiarity with a variety of local flaps and bone fixation techniques.^6^ Distant free fiaps have the added advantage of minimizing donor site burden or surgical trauma to an already compromised limb in comparison to local flaps. In addition, the provision of ample well-vascularized tissue can promote wound healing, minimize infection, and enable coverage of larger sized wounds.^1^

With advances in microsurgical techniques and instrumentation, free flap survival rates have risen from 79% to 96% with similar rates among most centers.^2^^,^^7^ With this increased proficiency in technique, there are a myriad of free flaps available to the reconstructive surgeon. The surgeon should be cognizant to try and utilize a flap with the longest vascular pedicle possible to ensure that the vascular anastomosis is outside the field of tumor resection and radiation. Muscle flaps such as the gracilis, rectus abdominis, and latissimus dorsi can bring a large amount of well-oxygenated tissue to a defect and obliterate a large volume of dead space. Given the increased size of these flaps, they can best be suited to cover larger defects of the upper extremity as well as exposed articular surfaces.^1^^,^^6^ Fasciocutaneous flaps are useful for upper extremity reconstruction as these flaps can provide thinner, less bulky flaps without the morbidity associated with muscle resection. Commonly used fasciocutaneous flaps are the radial forearm flap, scapular flap, and the ALT. Given their comparative decreased thickness, these flaps can be ideal for wrist and hand reconstruction.^1^

In an effort to reconstruct the vasculature as well as provide soft tissue coverage, a flow-through flap may be utilized. In this technique, the flap inflow arterial system not only provides perfusion to the transported flap but also provides a vascular link between the obliterated segment of vasculature. Thus, one requires a long vascular pedicle with visualization of both inflow and outflow systems if a flow-through flap will be utilized.^8^

The ALT fulfills many of the requirements of an ideal free flap.^2^ One of the primary advantages of the ALT flap is the reduced donor-site morbidity. Careful dissection and preservation of the vastus lateralis motor nerve as well as skeletonization of muscle perforators preserves muscle and maintains maximal quadriceps function.^4^ Additional advantages include a long pedicle with suitable vessel diameter as well as the availability of large amounts of skin.^9^ Finally, the ALT flap can be utilized as a flow-through flap with the pedicle bridging vascular gaps in extremity reconstruction.^3^^,^^10^

We present a case of a 76-year-old Caucasian woman with a recurrent cutaneous squamous cell carcinoma. Local flap coverage was not an option because of the extensive prior resections. Free flap coverage with a contralateral forearm-based flap was refused by the patient because of concern for morbidity to her dominant arm. A major reconstructive challenge of this case was the interruption of the superficial vascular arch following amputation of the ring and little fingers. Although retrograde flow was noted in the distal stump of the transected superficial arch, long-term maintenance of this vascularity was unknown. Loss of this retrograde flow into the residual ulnar artery could have led to loss of the patients remaining left hand. We believe that the case presented is truly unique as a large, composite defect with an obliterated palmer arch was created in an elderly, active patient and this anatomy was reconstructed in a 1-stage procedure with excellent long-term results.
